# Understanding the disparity of educational attainment: the role of socio-demographic and school-level factors on GCSE attainment in Northern Ireland.

**DOI:** 10.23889/ijpds.v7i3.1773

**Published:** 2022-08-25

**Authors:** Erin Early, Sarah Miller, Laura Dunne, John Moriarty

**Affiliations:** 1 UCL; 2 QUB

Educational attainment disparities across social groups remain at the forefront of contemporary UK society. Despite this, Northern Ireland reflects a somewhat different context to the rest of the UK due to its transition to a post-conflict society and its dually selective education system (academically and religiously). In Northern Ireland, post-primary (GCSE) attainment differences are often reported according to gender, religious affiliation and socio-economic background. However, due to the lack of available education data that encompasses a wide range of pupil- and school-level factors, discourse informed by the statistical testing of such factors has been limited. This study aims to overcome this current gap by examining the effects of socio-demographics, namely gender, religious affiliation and socio-economic background (through eight measures), and school-level factors on GCSE attainment, using the first linked administrative dataset for education in Northern Ireland. The data combined the household Census (2011) with the School Census (2010-2014) and School Leavers Survey (2010-2014) for the first time in Northern Ireland. To this end, this paper discusses data analytics of the study including data linkage, cohort size, constructed GCSE attainment measures, socio-demographic measures and school-level factors. The multilevel modelling (including interaction models) construction, execution and results will also be discussed. The paper concludes with a reflection upon whether the results of this analysis support existing literature in the Northern Ireland context and wider UK GCSE attainment trends.

